# Individual differences in strategy use and performance during fault diagnosis

**DOI:** 10.1186/s41235-020-00250-5

**Published:** 2020-10-23

**Authors:** Michael Shreeves, Leo Gugerty, DeWayne Moore

**Affiliations:** 1 Psychology Department, Clemson University, Clemson, USA; 2 Present Address: Arizona State University at Lake Havasu City, 100 University Way, Lake Havasu City, AZ 86403 USA

**Keywords:** Individual differences, Normative reasoning, Fault diagnosis, Diagnostic reasoning, Thinking dispositions

## Abstract

**Background:**

Research on causal reasoning often uses group-level data analyses that downplay individual differences and simple reasoning problems that are unrepresentative of everyday reasoning. In three empirical studies, we used an individual differences approach to investigate the cognitive processes people used in fault diagnosis, which is a complex diagnostic reasoning task. After first showing how high-level fault diagnosis strategies can be composed of simpler causal inferences, we discussed how two of these strategies—elimination and inference to the best explanation (IBE)—allow normative performance, which minimizes the number of diagnostic tests, whereas backtracking strategies are less efficient. We then investigated whether the use of normative strategies was infrequent and associated with greater fluid intelligence and positive thinking dispositions and whether normative strategies used slow, analytic processing while non-normative strategies used fast, heuristic processing.

**Results:**

Across three studies and 279 participants, uses of elimination and IBE were infrequent, and most participants used inefficient backtracking strategies. Fluid intelligence positively predicted elimination and IBE use but not backtracking use. Positive thinking dispositions predicted avoidance of backtracking. After classifying participants into groups that consistently used elimination, IBE, and backtracking, we found that participants who used elimination and IBE made fewer, but slower, diagnostic tests compared to backtracking users.

**Conclusions:**

Participants’ fault diagnosis performance showed wide individual differences. Use of normative strategies was predicted by greater fluid intelligence and more open-minded and engaged thinking dispositions. Elimination and IBE users made the slow, efficient responses typical of analytic processing. Backtracking users made the fast, inefficient responses suggestive of heuristic processing.

## Significance

Experts and novices often need to find (diagnose) the causes of specific problems, for example, when physicians diagnose illnesses, citizens understand the causes of global warming, or a couple figures out why their teenager’s grades are plummeting. We studied fault-diagnosis strategies like ruling out causes (elimination) and explaining the most effects with the fewest causes (inference to the best explanation or IBE). Because fault diagnosis is so widely applicable, understanding its underlying cognitive processes can lead to training that improves peoples’ ability to diagnose faults in a variety of physical systems and social situations. Prior research on training fault diagnosis strategies has taught either inefficient backtracking strategies or overly specific strategies (e.g., for a water supply system) that may not generalize to new situations. Our studies found that elimination and IBE minimize the number of diagnostic tests, while some backtracking strategies save time. This suggests that people should be taught a repertoire of strategies that fit different situations, e.g., elimination and IBE if tests are very expensive and backtracking for speed.

Because strategies like elimination and IBE are useful in understanding policy issues, our findings are relevant to teaching critical thinking to the public. We found that intelligence and positive thinking dispositions (e.g., open-mindedness, intellectual engagement) are positively associated with using elimination and IBE. Since thinking dispositions may be more trainable than intelligence, teaching thinking dispositions may be a more effective way to increase the use of elimination and IBE. These strategies can also be taught directly as part of education for critical thinking.

## Background

This project focuses on individual differences in two aspects of causal reasoning. In causal learning, people induce *general* knowledge about the strength and structure of causal relationships after observing large-sample covariation data (Griffiths and Tenenbaum 2009) or by making interventions and observing their effects (Bramley et al. 2017). In diagnostic reasoning, people use previously learned causal knowledge and a small number of observed events to make inferences about other *specific* events (Meder and Mayrhofer 2017a). In this paper, we focus on a particular kind of diagnostic reasoning known as fault diagnosis.

### Fault diagnosis

Fault diagnosis is an arguably general-purpose^1^ process that involves finding the causes that are producing specific abnormal effects in a system (symptoms). Fault diagnosis is common in equipment repair and medicine, but the reasoning used in fault diagnosis is applicable in other domains. Legal reasoning (Fenton et al. 2013) and some scientific argumentation (e.g., identifying causes of global warming) seek to identify the causes of specific observed events. Fault diagnosis is not just done by experts. Home care nurses sometimes need to diagnose faults on home medical devices and may have difficulty doing so (Lyons and Blandford 2018). If your laptop cannot get a Wi-Fi signal but your cell phone can, the working cell phone allows you to eliminate an absent Wi-Fi signal as the cause and localize the fault to your laptop. Gugerty (1989) found that many undergraduates used this elimination strategy when diagnosing a household electrical problem. Causal attribution in social situations (Morris and Larrick 1995) uses causal inferences like discounting, which is used in fault diagnosis.

Little lab-based research has been conducted on fault diagnosis, as most research has focused on experts working in complex, knowledge-rich domains, e.g., medical diagnosis (Patel et al. 2012). In this project, we used a fault diagnosis task that is more complex than many tasks used to study diagnostic reasoning but which does not require prior expertise and can be used in a laboratory setting. We focus on three fault diagnosis strategies: *backtracking* from the abnormal system output, *eliminating* potential faults that lead into normal system output, and *inference to the best explanation* (IBE). In IBE, people choose a causal explanation of a set of symptoms based on simplicity (minimizing the number of faults), coverage (maximizing the number of symptoms explained), and other factors (Lombrozo and Vasilyeva 2017).

### Individual differences in normative performance

Researchers may obtain an inaccurate picture of peoples’ cognition by focusing only on group averages, especially when individuals can complete a task using different strategies. Such differences have been shown in learning and memory (Estes 1956; Hemmer et al. 2015) and spatial cognition (Logie 2018). However, within research on reasoning, many of the early heuristics-and-biases studies focused on group averages and concluded that the *average* person fell short of normative standards on a variety of inductive (Nisbett et al. 1983; Tversky and Kahneman 1974) and deductive (Evans et al. 1983; Wason and Shapiro 1971) reasoning tasks.

More recent findings on individual differences have emphasized how subgroups of people vary from the group mean. First, a small group of participants reason normatively on many reasoning tasks, although most fail to do so. Second, the extent to which individuals reason normatively is positively correlated with fluid intelligence and thinking dispositions (e.g., open-mindedness), with each predictor contributing uniquely (Klaczynski and Lavalee 2005; Stanovich and West 1997, 1998; Toplak et al. 2014). In the current studies, we investigate these two research questions using the complex reasoning task of fault diagnosis.

Many cognitive science researchers define normative cognition using models at Anderson’s (1991) rational level of explanation, which describes cognitive functions in terms of optimal adaptation to goals given environmental constraints. Here, we use information gain as a metric for defining optimal or normative fault diagnosis. Information gain is a frequently used metric of normative performance in fault diagnosis (Navarro and Perfors 2011) and other information search tasks (Nelson 2005).

### Fluid intelligence and thinking dispositions

The ability to concurrently process and store information in working memory is strongly correlated with performance on a variety of reasoning tasks (Kyllonen and Christal 1990). Researchers have recently focused on a particular function of working memory—supporting inferences about hypothetical situations that are decoupled from perceptual representations of the world—as an important component of reasoning (Evans and Stanovich 2013; Oaksford and Chater 2012). Evans and Stanovich (2013) suggested that tests of fluid intelligence assess this function of working memory; Shipstead et al. (2016) provided evidence supporting this viewpoint.

Stanovich (2011, 2018) proposed that tests of thinking disposition assess the degree to which people can detect the need to override less-effortful thinking that relies on prior knowledge and switch to analytic, hypothetical thinking. Common thinking disposition measures include motivation and effort toward cognitive tasks (e.g., Typical Intellectual Engagement; Goff and Ackerman 1992) and openness to changing beliefs (e.g., Actively Open-Minded Thinking; Stanovich and West 1997). In this viewpoint, fluid intelligence assesses the capability for hypothetical thinking using working memory, while thinking dispositions assess the propensity to do this.

### Research questions

Our goal was to test whether the individual differences findings described above, which have been demonstrated primarily on simpler reasoning tasks, extend to complex causal reasoning tasks. We conducted three studies in which participants completed tests of fluid intelligence and thinking dispositions and a task where they diagnosed faults in physical systems. We focused on the strategies mentioned above—backtracking, elimination, and IBE—and considered two research questions related to individual differences. Are strategies that allow normative performance used less frequently than strategies not associated with normative performance (Q1)? Are strategies associated with normative performance used more frequently by people with higher fluid intelligence and thinking dispositions (Q2)? Also, to better understand the cognitive processes used in fault diagnosis, we investigated whether elimination, IBE and backtracking have characteristics of analytic or heuristic processing (Q3).

#### Causal learning task

In two of our studies, participants completed a causal learning task (Liljeholm and Cheng 2009) in addition to the fault diagnosis task. As noted above, learning causal models of the world and using these models to make useful inferences are two critical aspects of causal reasoning. This research design allowed us to pursue our research questions for both causal reasoning tasks and also to see whether the use of normative strategies was correlated across the two tasks. Due to length considerations, we were not able to present the findings from both tasks in this paper. We plan to present the causal learning findings and the cross-task correlations in a separate paper.

### Diagnostic reasoning

In causal learning, people learn a causal model describing the relationships among some causal and effect variables—including their structure and strength—by observing many co-occurrences of cause and effect variables (Lu et al. 2008) or by observing the effects of interventions they have selected (Bramley et al. 2017; Coenen et al. 2015). Peoples’ causal models are general in that they are applicable to many situations. Many studies of diagnostic reasoning, including ours, assume that participants have already learned a causal model that describes a particular situation, based on either in-study training or prior expertise. Then, participants observe the state of a small number of variables in the model and make inferences that update their beliefs about the state of other model variables. We consider diagnostic reasoning to be a broad category that includes the following: (1) single-step inferences between directly linked variables, including diagnostic (effect-to-cause) and predictive (cause-to-effect) inferences; (2) multistep inferences such as inference chaining and discounting (Waldman et al. 2008); and (3) higher-level processes like fault diagnosis and forecasting. (In this paper, the term diagnostic *inference* refers to effect-to-cause inferences between directly connected variables, while diagnostic *reasoning* is a much broader term, as described here.)

#### Simpler diagnostic reasoning tasks

Much of the research in a recent review of diagnostic reasoning (Meder and Mayrhofer 2017a) used lab-based tasks with relatively simple causal structures, i.e., a few causes and fewer than a dozen effects. Researchers often assume that, given these simpler structures, people make diagnostic inferences in a *quantitative* fashion (e.g., Meder and Mayrhofer 2017b; Waldmann et al. 2008). For example, the ability to accurately estimate the posterior probability of a cause after observing its effect correlates positively with fluid intelligence and thinking dispositions (McNair and Feeney 2015; Sirota et al. 2014).

#### Handling complexity

Realistic fault-diagnosis problems often have complex causal structures. For example, based on verbal protocols given by physicians as they diagnosed realistic cases, Patel et al. (1990) created causal networks showing physicians’ predictive and diagnostic inferences. One physician’s network contained 12 nodes representing evidence from the case and 24 causal nodes representing physiological conditions (including the correct diagnosis). Another physician’s network contained four pieces of evidence and 15 causal nodes. In each problem of our fault diagnosis task, 25–35 potential faults were present, and participants could gather 40–60 pieces of evidence.

Research has shown that as the complexity of decisions increases (e.g., more alternatives or attributes), participants shift from evaluating alternatives using quantitative reasoning (e.g., weighted additive strategy) to *qualitative* strategies like elimination by aspects, which iteratively eliminates choices (Payne and Bettman 2004). Studies of diagnosis by physicians and nurses suggest that they use qualitative reasoning as well, e.g., classifying diseases as being in or out of a set of potential causes instead of assigning each disease a graded probability of causing the symptoms (Eddy and Clanton 1982; Johnson et al. 1982; Rossi and Madden 1979). Also, evidence indicates that children (Schulz and Sommerville 2006) and adults (Austerweil and Griffiths 2011; Lu et al. 2008; Yeung and Griffiths 2015) often assume qualitative, deterministic causes (either strong or absent) even in complex domains where causal strength varies continuously. Sloman and Lagnado (2015) have highlighted the importance of qualitative reasoning in causal reasoning. In the current studies, we used a deterministic task and focused on how qualitative reasoning might be used in fault diagnosis.

### Fault diagnosis

**Fig. 1:**
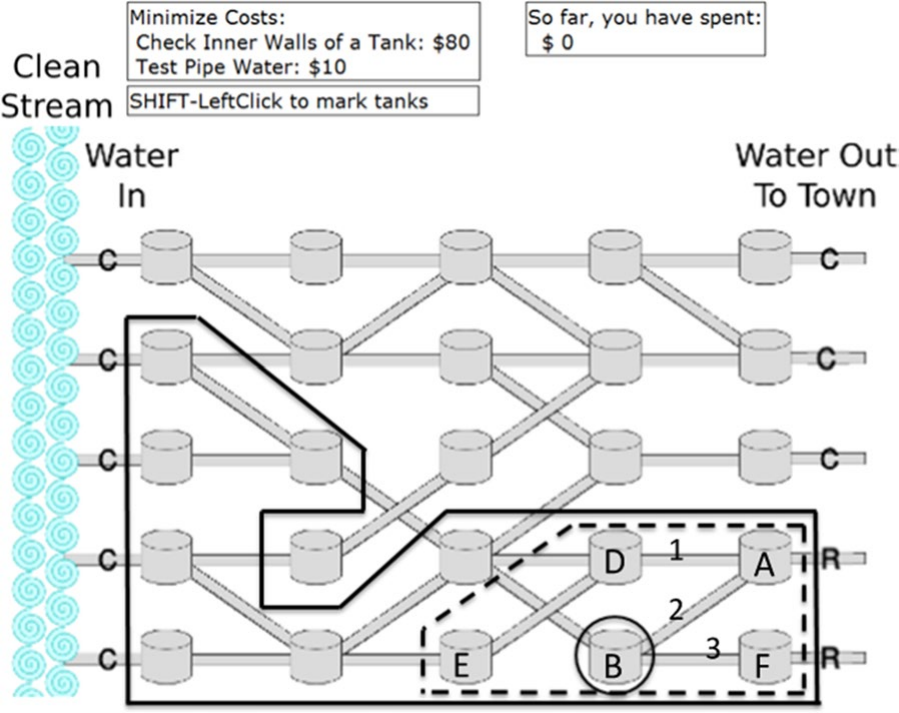
Initial display (before any tests made) for a fault diagnosis problem, showing a network of water storage tanks with water flowing from left to right and observations of clean (C) and rusty (R) water flowing in pipes. The overlays (which were not shown to participants) show the reduced fault set after using general backtracking (solid straight lines), elimination (dashed lines), and IBE (circle) based on the network outputs at the beginning of the problem. The pipe numbers and tank letters were not shown to participants. The rusty tank is circled

Our participants solved problems like in Fig. 1, which shows a network of water storage tanks through which water flowed from left to right. At the start of each problem, the display showed whether clean (C) or rusty (R) water was flowing through the network input and output pipes. The network is taking in clean water but outputting rusty water because a tank is rusty. The goal of the participants was to find the rusty tank. They did this by making diagnostic tests (testing a pipe revealed whether it contained clean or rusty water) and submitting diagnoses (checking a tank revealed whether it was clean or rusty inside). Results of tests and incorrect diagnoses (a C or R by a pipe or tank) remained on the display. Participants made tests and diagnoses until they diagnosed the rusty tank. They were instructed that (1) pipe tests and tank checks were costly and should be minimized, (2) only one tank was rusty, and (3) rusty tanks had very strong effects (i.e., deterministic causes). Participants were incentivized to observe the cost constraint by imaginary monetary costs and by delays between the time when they clicked on a pipe or tank and when the test result appeared (2.5 s delay and $10 for pipe tests; 5–12.5 s delay and $80 for tank checks). The cumulative amount of money spent on each problem was updated after each test or check. After diagnosing the rusty tank, participants received feedback about how well they had met the cost constraint. Participants could place visual markers on tanks, which were intended to reduce the memory load of using diagnosis strategies. “Appendix 1” shows the networks used in the studies. Similar tasks have been used by Carlson et al. (1992), Kostopoulo and Duncan (2001), and Ham and Yoon (2007).

According to Nelson (2005), fault diagnosis is an example of the general inductive problem where people have a set of hypotheses, and data relevant to the hypotheses are currently available or potentially available from queries (e.g., diagnostic tests). In fault diagnosis, the causal hypotheses are that some system components could be faulty. The task involves efficiently selecting diagnostic tests, which allow observing the effects of potential faults and then updating beliefs about hypotheses based on observed effects. Many studies have focused on the test selection component of this inductive problem (Klayman and Ha 1987; Oaksford and Chater 1994, 2003; Ruggeri and Lombrozo 2015). In the current studies, we focused on the belief-updating component, i.e., how participants updated their beliefs about causal hypotheses using observations, such as observing rusty versus clean water in pipes. In order to highlight to participants the importance of using efficient belief-updating strategies, in each study participants saw two problems like Fig. 1, where using elimination and IBE allowed diagnosing the rusty tank without any diagnostic (pipe) tests, based only on belief updating using the initial observations.

Researchers have distinguished two types of hypothesis testing (Ruggeri and Lombrozo 2015). The more efficient *constraint seeking* involves making diagnostic tests that reveal the effects of faults and then updating (narrowing) the fault set based on the observed effects. *Hypothesis scanning*, which focuses on root causes instead of effects, involves making diagnoses of potential faults. This approach is inefficient because it only narrows the fault set by one potential fault per diagnosis and provides no new observations that allow updating the fault set. Given our focus on belief updating, we set the time and money costs for diagnoses to be much higher than for diagnostic tests of pipes in order to motivate participants to minimize the number of diagnoses and use pipe tests as their problem-solving operator. (In Study 1, where the delay after unsuccessful diagnoses was 5 s, a few participants made too many diagnoses. This tendency was reduced by setting this delay to 12.5 s in Study 2 and Study 3.) In the following, we describe the belief-updating strategies that we studied. All of these strategies narrow the fault set by eliminating some faults from consideration, although some strategies do this more efficiently than others.

#### General backtracking

In backtracking, reasoners update the fault set (which initially contains all tanks) at the outset of the problem by first making one-step diagnostic inferences from the observations of abnormal system state (rusty water), which generates the hypotheses that a tank outputting rusty water could be rusty or any pipe leading into it could carry rusty water. Instead of testing these hypotheses about pipes with diagnostic tests, reasoners make diagnostic inferences from them. This process is repeated recursively until no more inferences can be made. These chains of diagnostic inferences create a set of potential faults that we call the backtracking set and eliminate tanks that that do not lead into rusty water. Figure 1 shows the initial backtracking set, before any diagnostic tests have been made.

After the initial update, reasoners test pipes that directly connect tanks within the current backtracking set until they find a rusty water result and then update the backtracking set again. This test–update cycle is repeated until a diagnosis can be made, i.e., when a tank is identified with all clean water inputs and rusty water outputs. Backtracking is inefficient because it ignores useful information—observations of normal system state (clean water). We call this strategy general backtracking to distinguish it from a variant of backtracking described later.

Updating the backtracking set after each rusty-water test result means that tanks that were in the previous backtracking set but are not causally upstream of the latest rusty water observation are eliminated from the fault set even though they lead into rusty water and could be rusty. For example, in Fig. 1, after pipe 2 has been tested and found to carry rusty water, the new backtracking set contains tank B and all tanks upstream of it, while tanks A, D, E and F are eliminated even though they could be rusty if there were multiple rusty tanks. This second type of elimination during backtracking depends on the single fault assumption. Whether participants using backtracking are consciously eliminating these tanks based on the single fault assumption or merely focusing on making diagnostic, upstream inferences until they find definitive evidence for a rusty tank is not clear.

#### Practiced elimination

At the outset of the problem, reasoners using elimination make recursive diagnostic inferences (without diagnostic testing) as in general backtracking but from the clean instead of the rusty water observations. This creates the initial elimination set (Fig. 1). Then, they test a pipe directly connecting two tanks in the current elimination set and update the set by eliminating tanks causally upstream of a clean water result and, as in general backtracking, not upstream of a rusty water result. This procedure is iterated until the faulty tank is identified. Although all the updating strategies we discuss involve elimination, only the strategy we call elimination rules out potential causes that predict effects that are disconfirmed by observations of *normal system state*. This approach is consistent with the idea of eliminating or ruling out hypotheses in medical diagnosis, which is discussed below. This strategy is called practiced elimination to distinguish it from a variant of elimination discussed below.

#### IBE

Researchers have identified a number of “explanatory virtues” (Lipton 2004) that reasoners use to choose the best explanation of some effects. These explanatory virtues include explaining more effects (coverage) (Johnson et al. 2014), having fewer root causes (simplicity) (Lombrozo 2007; Pacer and Lombrozo 2017), and being more coherent with background information (Koslowski et al. 2008). As its name implies, IBE also involves an inference procedure that evaluates the quality of various explanations in light of sometimes conflicting explanatory criteria. In fault diagnosis, starting with the current elimination set, the coverage and simplicity criteria allow eliminating potential faults that do not explain all of the observations of abnormal system state (symptoms). One inference procedure that accomplishes this is to make predictive inferences from each potential fault in the elimination set and eliminate those that are not causally upstream of all of the symptoms. This procedure is similar to one used in a machine learning model of IBE based on causal information flow (Pacer et al. 2013). Note that in this viewpoint, IBE involves testing potential faults by making hypothetical, predictive inferences.

By telling participants that there was only one rusty tank per network, we primed them to use IBE by revealing that a simple, one-fault explanation could cover or explain all the symptoms. However, we did not tell them how to make the inferences to identify the best explanation. Thus, if we find participants who always make tests and diagnoses within the IBE set, we cannot make the strong claim that they are using IBE without any aid, but we can claim that they are exhibiting the inferences that are part of IBE.

#### Examples

**Fig. 2:**
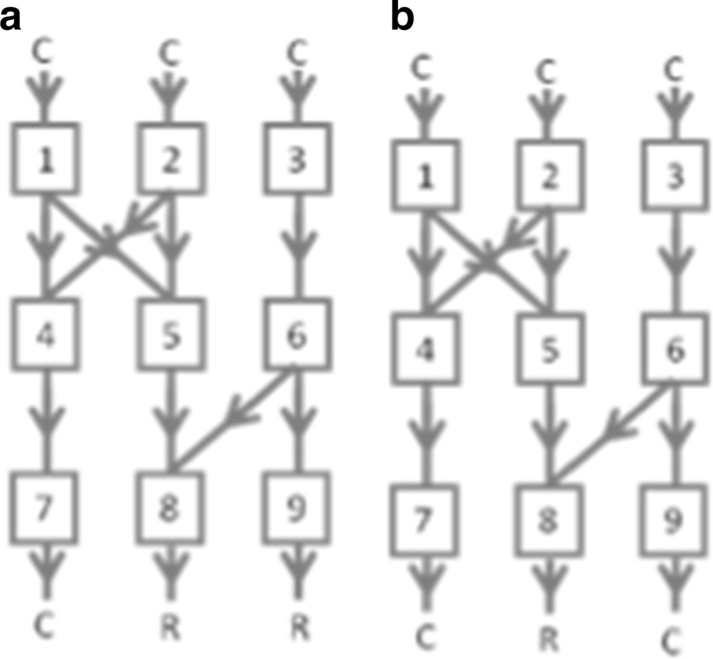
Tank networks with two (panel A) and one (panel B) rusty network outputs. Water flows from top to bottom. “R” means rusty water in a pipe. “C” means clean water

In Fig. 2a (where tank 6 is rusty), the general backtracking set is tanks 1, 2, 3, 5, 6, 8, and 9; the practiced elimination set is 3, 5, 6, 8, and 9; and the IBE set is 3 and 6. If someone tested the pipe connecting tanks 1 and 5, i.e., pipe 1–5 (with result C), and then tested pipe 2–5 (C), 6–8 (R), and 3–6 (C), these four tests would be evidence for general backtracking but not elimination or IBE since some of the tests are outside the elimination and IBE sets. If someone tested pipe 5–8 (C), 6–8 (R), and then 3–6 (C), this would be evidence for elimination but not IBE. People using IBE would only test pipe 3–6 (C), as this test isolates the fault. See the supplementary materials for demonstrations of the strategies.

#### Stepwise backtracking

In *stepwise backtracking*, reasoners make one-step diagnostic inferences from the observations available at the outset of the problem, and then (unlike in general backtracking) immediately test the pipes leading into the tank outputting rusty water. If a pipe test reveals rusty water, the strategy is applied recursively from this result. In Fig. 2b (with tank 5 rusty), the following test sequence exemplifies stepwise backtracking: 6–8 (C), 5–8 (R), 1–5 (C), and then 2–5 (C). Thus, testing starts at the network rusty output and moves upstream.

#### Discovering elimination

Here we describe more exploratory reasoning that may allow reasoners to transition from backtracking to elimination. In Fig. 2b (general backtracking set: tanks 1, 2, 3, 5, 6, and 8), suppose a reasoner using general backtracking hypothesized that pipe 3–6 contains rusty water. Instead of making a diagnostic test of this pipe, the reasoner could make the predictive inferences that pipes 6–9 and the output for 9 contain rusty water. Since the final prediction is disconfirmed by observed evidence, pipes 3–6, 6–9, and 6–8 must contain clean water, and tanks 6 and 9 must be clean. A sequence of hypothetical predictive inferences like this could lead a reasoner to discover the elimination strategy. After practicing this discovery strategy, participants could develop the more efficient elimination strategy described earlier, in which they immediately rule out tanks leading to clean water without making predictive inferences. In the following, the term elimination refers to the practiced version.

#### Prior research on belief updating strategies

Gugerty (2007) found that college students used mostly backtracking on a version of the current task and showed little use of elimination. Johnson et al. (1982) provided evidence that medical students, but not physicians, used stepwise backtracking during diagnosis. Carlson et al. (1992) found that undergraduates trained in stepwise backtracking followed it more than an untrained group but that training did not reduce testing costs. Patel et al. (1990) and Johnson et al. (1982) documented instances of physicians using practiced elimination on patient cases.

We noted above that hypothetical thinking based on predictive inferences was used in discovering elimination and in IBE. This approach is important because hypothetical thinking has been implicated as a key part of fluid intelligence and analytic thinking (Evans and Stanovich 2013), which relate to two of our research questions. Gugerty (2007) found that participants who initially used only backtracking increased their use of elimination (relative to control participants) when trained to test fault hypotheses by predictive reasoning instead of diagnostic tests. Patel et al. (1990) and Johnson et al. (1982) observed physicians ruling out hypotheses that conflict with evidence by making predictive inferences instead of diagnostic tests. Finally, IBE has been observed in field studies of medical diagnosis (Eddy and Clanton 1982; Kassirer 1989).

#### Which strategies are normative?

We defined normative performance for updating strategies in terms of maximizing information gain. Normative performance is a common metric for measuring peoples’ efficiency at posing questions to gather information, including the test selection component of fault diagnosis. Nelson (2005) showed that information gain is at least as efficient as other metrics (e.g., diagnosticity) for quantifying performance on question-posing tasks. For example, Navarro and Perfors (2011) proved that the half-split strategy—selecting the test that comes closest to eliminating half of the hypotheses—is normative in the sense that it maximizes information gain and minimizes the number of tests. Using a task where participants asked yes–no questions to determine the cause of an event, Ruggeri and Lombrozo (2015) found a developmental shift between ages 7–18, whereby older participants asked questions that more effectively narrowed the search space, resulting in higher information gain and fewer tests. Bramley et al. (2017) used information gain to define normative performance at selecting interventions to learn the causal structure of a system.

However, once people have selected an efficient diagnostic test, they must appropriately update the hypothesis set to realize any information gain. Consider a reasoner who conducts a half-split pipe test that reveals clean water. If this person is unaware that tanks upstream of clean water can be eliminated, she might fail to update the fault set appropriately, resulting in no information gain. For deterministic problems where no further narrowing can be accomplished by IBE, de Kleer and Williams (1987) proved that elimination minimizes the size of the fault set. It is important to note that the fact that elimination minimizes tests is an emergent property that falls out of the process of making diagnostic and predictive inferences from all of the available observations. When multiple abnormal system outputs are present, elimination and IBE can be used together. To the extent that the single-fault constraint is warranted, elimination followed by IBE is normative and minimizes the size of the fault set because it uses all the information and constraints that can reduce this set.

Thus, to minimize diagnostic tests during fault diagnosis, participants should use elimination and, if needed, IBE for belief updating and half split for test generation. However, given the size and structure of the networks used in our study, the updated fault sets after use of elimination and IBE were usually so small that half-split could not be used (i.e., they contained three or fewer pipes). We asked participants to minimize the costs of diagnostic tests and diagnoses, which required minimizing the number of tests, because we wanted to assess their capabilities for normative fault diagnosis. In preliminary testing, when there was no delay after pipe tests, many participants seemed to be minimizing time use rather than costs, as they used mostly backtracking and made many very fast tests. The 2.5 s delay after each pipe test was implemented to encourage them to minimize tests.

### Measuring strategy use

Elimination use and backtracking were measured on blocks of five to nine network problems that had one rusty output so that IBE could not be used. IBE use was measured on a separate block of five to nine problems that had two rusty outputs. The sequence and timing of pipe tests, tank checks, and markers placed was recorded for each problem. We used both types of diagnostic *actions*—pipe tests and tank checks—to measure elimination and IBE use. For each participant, strategy use variables were calculated for each network problem and then averaged over problems in a block.

#### Elimination

Since almost all participants confined their actions to the general backtracking fault set, the elimination use variable was designed to measure the extent to which participants went beyond backtracking to use elimination on a single problem. We measured elimination use based on how frequently participants’ actions were within the set of potentially rusty tanks identified by this strategy. The percentage of elimination actions (%ElimActions) was the percentage of the total actions for a network that were in the current elimination set, which was updated after each test. However, since the elimination set is always a subset of the general backtracking set (e.g., Fig. 1), a participant using only backtracking will have some actions fall within the elimination set by chance. Therefore, actions in the elimination set do not unambiguously indicate elimination use. Accordingly, we only gave participants credit for using elimination if their percentage of elimination actions was above the percentage that would be expected for participants using backtracking. The chance percentage of actions falling within the elimination set given use of stepwise backtracking (chance%ElimActions) was estimated for each problem by averaging 10,000 runs of a simulation that diagnosed the fault using the stepwise backtracking strategy and calculated the percentage of actions within the elimination set. Then, to calculate *Elimination Use* for a problem, %ElimActions was corrected for chance:

1 $${\text{Elimination use}} = \frac{{\% {\text{ElimActions}} - {\text{chance}}\% {\text{ElimActions}}}}{{100 - {\text{chance}}\% {\text{ElimActions}}}}$$

Thus, elimination use measured how consistently an individual used elimination on a problem beyond the level expected from using only stepwise backtracking. Elimination use would be 100 for participants who used elimination for all actions on the problem and 0 (on average) for participants who always used stepwise backtracking. (The chance percentage of elimination tests based on stepwise backtracking is higher than the percentage based on general backtracking. Thus, using stepwise backtracking as the baseline yields a more conservative estimate of elimination use.)

#### IBE

Because the use of IBE without elimination will not minimize the fault set, we only gave people credit for using IBE if they also used elimination. Because the IBE set is always a subset of the elimination set, measuring IBE use presents the same problem as measuring elimination. Therefore, as for elimination, we only gave participants credit for using IBE if they made more tests in the IBE set than would be expected for someone using elimination but not IBE. The percentage of IBE actions (%IBEactions) was the percentage of the total actions for a network that were in the updated fault set based on using elimination and IBE. The chance percentage of actions falling within the IBE set for a person using elimination but not IBE was calculated for each network problem by simulation. Thus,

2 $${\text{IBE use}} = \frac{{\% {\text{IBEactions}} - {\text{chance}}\% \,{\text{IBEactions}}}}{{100 - {\text{chance}}\% \,{\text{IBEactions}}}}$$

Participants who used elimination and IBE for all actions on a problem would score 100 on IBE use, and those who used only elimination would score around 0.

The elimination use and IBE use variables describe how frequently individuals used these strategies. However, since the elimination set was a subset of the backtracking set, the percentage of general (or stepwise) backtracking actions cannot be used directly to characterize how frequently individuals used these strategies. In the results section, we describe how we measured the backtracking strategies and how we classified individual participants in terms of whether they *consistently* used any of the four strategies across all of the problems.

### Hypotheses and analyses

#### Infrequent versus modal strategies (Q1)

We expected that elimination and IBE would be used by a small percentage of participants, with backtracking being the modal strategy. Although this is a descriptive question with an imprecise criterion, the pattern of infrequent normative performance has been found for resisting belief bias in syllogistic reasoning and the Wason selection task (Stanovich and West 1998). Also, these data are important in understanding whether some individuals can achieve consistent normative performance on reasoning tasks.

#### Predictors of strategy use (Q2)

Our description of the elimination and IBE strategies suggested that these strategies rely on hypothetical thinking using working memory. Therefore, following Evans and Stanovich’s (2013) assumption that fluid intelligence and thinking dispositions assess the capability and propensity, respectively, to engage in this kind of working-memory intensive thinking, we hypothesized that fluid intelligence and thinking dispositions would correlate positively with elimination use and IBE use, with each predictor accounting for unique variance in using these strategies.

#### Heuristic versus analytic processing (Q3)

We also evaluated whether elimination, IBE, and backtracking involve heuristic or analytic processing. This evaluation was done in a post-hoc manner, without advancing a hypothesis. Analytic processing involves heavy use of working memory and exhaustive information processing, while heuristic processing involves processing environmental cues based on prior knowledge using mental shortcuts (Dreschler et al. 2014; Evans and Stanovich 2013). Elimination and IBE seem to have characteristics of analytic processing, as they require making and maintaining many inferences in working memory and using all the available evidence. Both backtracking strategies have characteristics of heuristic processing, as they focus primarily on salient environmental cues (rusty water), and because they ignore useful evidence from clean water, make fewer inferences. Thus, elimination and IBE should yield more accurate but slower performance compared to backtracking.

### Contribution

#### Individual differences

Research on causal reasoning has begun to address individual differences in causal learning strategies (Bramley et al. 2017; Bramley et al. 2015; Buehner et al. 2003; Coenen et al. 2015). However, these studies did not study fault diagnosis, and they did not investigate the cognitive correlates of reasoning strategies. Individual differences studies that have focused on diagnostic reasoning have tended to use tasks that are much simpler than fault diagnosis (McNair and Feeney 2015; Sirota et al. 2014). In contrast to these studies, the current project uses two causal reasoning tasks—fault diagnosis and causal learning—to investigate individual differences in the use of normative versus non-normative strategies and the cognitive correlates of normative strategy use. We are not aware of individual differences research that has investigated peoples’ performance on two causal reasoning tasks.

#### Cognitive processes in fault diagnosis

Our fault diagnosis task differs from other lab tasks used to study diagnostic reasoning in a number of ways. Most lab-based diagnostic reasoning tasks involve simpler causal structures than the fault diagnosis task. Also, many diagnostic reasoning tasks require participants to make a single, explicit, quantitative judgment on each problem (e.g., posterior probability), whereas in the fault diagnosis task, participants make multiple realistic actions (diagnostic tests) and, to perform effectively, must do Bayesian updating of their problem knowledge after each test.

Few studies have investigated the higher-level cognitive processes (e.g., strategies) used in fault diagnosis and diagnostic reasoning. Studies using fault diagnosis tasks similar to ours (Carlson et al. 1992; Kostopoulo and Duncan 2001; Ham and Yoon 2007) have evaluated how training methods affect diagnostic performance but have not measured participants’ frequency of using particular strategies. Also, we are not aware of studies that investigated whether particular fault diagnosis strategies involved analytic versus heuristic processing.

### Empirical studies

Studies 2 and 3 can be considered replications of Study 1; therefore, we present the results of the studies together. Here we describe minor variations across the studies. In Study 1, fluid intelligence was measured by SAT and ACT scores and thinking dispositions by open-mindedness (Stanovich and West 1997). A limitation of Study 1 was that only one measure was used for each predictor variable. In studies 2 and 3, we added two fluid abilities tests, verbal analogies, and (because the fault diagnosis task seemed to have a spatial component) a spatial reasoning test. In Study 2, we added intellectual engagement as a thinking dispositions test (Goff and Ackerman 1992). The main focus of Study 3 was to test a hypothesis related to the causal learning task. Because this created time limitations, we did not measure thinking dispositions in Study 3. For studies 2 and 3, because each predictor was measured using multiple tests, we used structural equation modeling (SEM) with latent variables representing fluid intelligence and thinking dispositions. This approach allowed us to assess the reliability of our predictors in the same causal model as our reasoning outcomes. Also, SEM identifies the unique variance accounted for among all observed variables, giving a more reliable estimate of all relationships involved than would be obtained by aggregating predictors.

Participants completed 18 fault diagnosis problems in Study 1 and ten fault diagnosis problems in Studies 2 and 3. To encourage participants to use mainly diagnostic (pipe) tests, the delay after submitting an incorrect diagnosis was increased from 5 s in Study 1 to 12.5 s in Studies 2 and 3. Finally, the instructions for the fault diagnosis task were improved across the studies, as described below.

## Methods

### Participants

The participants in all studies were Clemson University students and were compensated with course credit. Study 1 had 79 participants (69% female; age *M* = 19.2, SD = 1.1). Data for three participants were excluded, as explained later. In Study 2, for structural equation modeling, the two latent, predictor variables (intelligence and dispositions) were parsimonious with only five indicators and moderately high loadings. Following procedures in Maxwell (2000), we used a priori estimates for moderate correlations (*r* = 0.30) between the latent variables and between the latent variables and the outcomes. Power of 0.80 for the multiple *R*^2^ yields a sample size of approximately 65 and a sample size of approximately 145 for the unique effect of each predictor. Study 2 had 106 participants (66% female; age *M* = 19.2, SD = 1.1). Computer errors resulted in eight participants missing fault diagnosis data, leaving 98 participants. In Study 3, four of the 111 participants were excluded for not finishing in the time allotted for the session, leaving 107 participants (53% female; age *M* = 19.4, SD = 1.1). Fault diagnosis task data for two participants were dropped, as described later.

### Design

A correlational design tested whether participants’ frequency of using elimination, IBE, and backtracking was correlated with performance differences and with differences in fluid intelligence and open-mindedness. Descriptive analyses evaluated frequency of strategy use.

### Materials and tasks

The fault diagnosis problems were presented via computer. Figure 1 shows the display at the start of a problem. A text box shows problem constraints (costs of pipe tests and tank checks). While solving a problem, participants clicked on a pipe or tank to test it, and after a delay of 2.5 s for pipes and 5–12.5 s for tanks, they received feedback superimposed on the object (“C” for clean water in a pipe or a clean tank; “R” for rusty). After each test or check, the total amount of money spent on the current problem was updated in a text box. Participants could place circular markers around tanks by shift-clicking. After finding and checking the rusty tank, participants received feedback, e.g., “You spent $120; a good amount of money to spend on this problem is $80.” The spending goals were based on always using elimination and, when applicable, IBE and half split.

In all studies, the task training used graphics and text to convey how the networks, tanks, pipes, and markers worked; the monetary and temporal testing costs; the feedback concerning money spent; and a request to minimize monetary costs. The training also noted that each tank network had one rusty tank, each network had either one or two pipes outputting rusty water, and water from the rusty tank would not become diluted downstream. Following the training, participants completed three comprehension questions, including one about how many rusty tanks each network had, with the correct answer given as feedback. In Study 3, some information about how the tanks worked was repeated, comprehension questions were added, and questions were repeated if answered incorrectly.

Following training, participants completed three practice problems and 18 (Study 1) or ten (Studies 2 and 3) experimental problems. The first half of the experimental problems had one rusty network output; the last half had two.

#### Intelligence measures

For measures of fluid intelligence, participants in studies 1 and 2 self-reported their SAT and/or ACT composite scores. In Study 3, participants gave permission for retrieval of their SAT and/or ACT scores from university records. ACT scores were converted into SAT equivalents. In studies 2 and 3, the speeded (5 min) 18-item verbal analogies test was taken from the AFOQT verbal analogies test (Berger et al. 1990). In Study 2, the spatial test was the 32-item arrow grammatical reasoning task (Kyllonen and Christal 1990). Because it showed poor psychometric properties, grammatical reasoning was replaced in Study 3 by paper folding (Ekstrom et al. 1976). Frey and Detterman (2004) showed strong correlations between SAT and measures of fluid intelligence (Raven’s Advanced Progressive Matrices, *r* = 0.72) and intelligence (*g* factor from the Armed Services Vocational Aptitude Battery, *r* = 0.86). Kyllonen and Christal (1990) found that verbal analogies and arrow grammatical reasoning loaded highly on fluid intelligence. Kane et al. (2004) found the same for verbal analogies and paper folding.

#### Thinking disposition measures

Open-mindedness was measured with the 41-item Actively Open-minded Thinking scale (Stanovich and West 1997). In Study 2, intellectual engagement was measured with the 60-item Typical Intellectual Engagement scale (Goff and Ackerman 1992).

### Procedure

In Study 1, participants completed the fault diagnosis task and then the Actively Open-Minded Thinking and SAT-ACT questionnaires. Sessions lasted about an hour. In studies 2 and 3, participants completed the fault diagnosis and causal learning tasks in random order. Then, they completed the tests measuring predictor variables (in Study 2, intelligence and thinking dispositions measures in random order; in Study 3, intelligence measures in random order). Sessions lasted approximately 1.5 h.

## Results and discussion

In Study 1, three participants were excluded from data analysis before inferential analyses were conducted, leaving 76 participants. One participant showed very low use of elimination, IBE, and general backtracking and was a statistical outlier on elimination and IBE use. Two participants tested far too many tanks on each problem (and were statistical outliers on this variable), which made it hard to measure their strategy use. In Study 3, data for two participants were excluded because they did not follow instructions to minimize tank checks (and were statistical outliers on this variable), leaving 105 participants. Nine participants in Study 2 and two participants in Study 3 had missing SAT and ACT scores; these scores were imputed, as explained later.

### Frequency of strategy use (Q1)

The frequency of elimination and backtracking (general and stepwise) was calculated using the first block of nine or five problems where only a single rusty network output was present and IBE could not be used. The frequency of IBE use was calculated using the second problem block where two rusty outputs were present. In the introduction, we described how we measured the extent to which participants used elimination and IBE on individual problems (see Eqs. 1, 2). These two variables ranged from approximately 0, which indicated no use of a strategy, to 100, which indicated constant use. These variables were averaged across the experimental problems in the first and second block to estimate overall elimination and IBE use, respectively, for each participant.

**Fig. 3:**
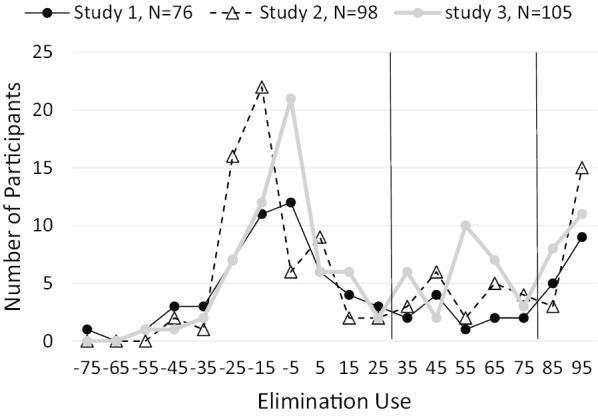
Histograms of elimination use for studies 1, 2, and 3. Vertical lines are used to define consistent use of backtracking (either form) and elimination (see text)

Across the three studies, elimination use was low, with mean values (across all participants in a study) ranging from 17 to 28 on a scale from near 0 to 100, and showed high variability across participants (see Table 1). Figure 3 shows the reason for this variability. Elimination use was distributed approximately bimodally with modes near 0 and 100. Because the elimination use variable corrects for chance (i.e., for tests in the elimination set that are consistent with backtracking), the roughly normal distribution around the mode near 0 (from − 35 to 25) is the degree of elimination use that would be expected for participants using only backtracking. The mode near 100 represents participants using elimination. A few participants showed high negative elimination use (< − 40), which was probably due to their selecting tests using the less-efficient general backtracking strategy or sometimes testing outside the backtracking set. IBE use was approximately normally distributed and low, with means ranging from 22 to 30 across the studies. Elimination and IBE use were highly correlated, Study 1: *r* = 0.74, *p* < 0.001, 95% CI [0.62, 0.83]; Study 2: *r* = 0.70, *p* < 0.001, 95% CI [0.58, 0.79]; Study 3: *r* = 0.75, *p* < 0.001, 95% CI [0.65, 0.82].

**Table 1:** Performance on the calculated strategy variables

	Study 1 (*N* 76)		Study 2 (*N* 98)		Study 3 (*N* 105)	
	*M*	SD	*M*	SD	*M*	SD
Elimination use (~ 0–100)	16.7	46.2	21.3	46.7	27.8	43.4
IBE use (~ 0–100)	30.1	38.5	21.7	43.7	24.7	43.4
% General backtracking actions	97.4	4.4	97.3	5.1	97.0	6.2
% Stepwise backtracking tests	77.1	16.1	72.1	20.8	72.1	22.8

Means were averaged across the 10–18 experimental problems each participant completed and then across participants

#### Identifying consistent strategy use

Based on the distributions of elimination use for all three studies, we defined individuals who consistently used elimination as those with average elimination use above 80. Consistent IBE use was defined using the same threshold. Determining whether a participant was using the general and stepwise backtracking strategies was more difficult. To start, we calculated the percentage of fault-diagnosis actions on a problem that were within the updated general or stepwise-backtracking set (see Table 1). The key difficulty in measuring backtracking use stems from the fact that all diagnosis actions following from the elimination strategy are also consistent with backtracking. Therefore, some participants who showed a very high percentage of actions within the general or stepwise backtracking sets were actually using elimination consistently (and possibly IBE), not backtracking.

Table 1 shows that all participants made almost all of their actions within the general backtracking set—suggesting that all participants understood backtracking—and none did random testing among all network tanks. Because all tests in the elimination set are within the general backtracking set, participants who were *not* using elimination consistently were either using one of the backtracking strategies or transitioning from backtracking to elimination. Transitioning could occur by the strategy we described in the introduction as discovering elimination, although it could occur in other ways. Therefore, based on the distributions in Fig. 3, we classified participants with low elimination use (below 30) as using either form of backtracking and participants with elimination use between 30 and 80 as transitioning from backtracking to elimination. Participants in the backtracking group were classified as using stepwise backtracking if their percentage of stepwise backtracking tests was greater than 90%; otherwise they were classified as using general backtracking.

Table 2 shows the percentage of participants in each study that were classified as consistently using different strategies by this scheme. Note that the first four strategy categories are defined to be mutually exclusive. Elimination was used consistently by 18% of participants in each study. Some form of backtracking was the modal strategy, as it was used by 55–67% of participants. Stepwise backtracking was less frequent than general backtracking. From 15 to 27% were transitioning from backtracking to elimination. Thus, the hypothesis that elimination use would be infrequent and backtracking more common was supported, even if the transitional participants are considered to be part of the elimination group. Consistent IBE use was also infrequent (8–14%), as hypothesized.

**Table 2:** Percentages of participants who used strategies consistently

strategy	Study 1 (*N* 76) (%)	Study 2 (*N* 98) (%)	Study 3 (*N* 105) (%)
Elimination	18	18	18
Transition	15	20	27
General backtracking	45	47	38
Stepwise backtracking	22	14	17
Either backtracking	67	61	55
IBE	8	11	14

#### Marker use

How participants used markers provides evidence for strategy use that is independent of the evidence above, which is based on the locations in the networks where participants conducted pipe tests and tank checks. As discussed in the introduction, elimination and IBE seem to put heavier loads on working memory than backtracking because they require maintaining and updating information about which tanks have and have not been eliminated. At the other extreme, stepwise backtracking seems to create minimal working memory load because it involves maintaining a very small set of potential faulty tanks. Since the markers could be used to reduce memory load, marker use was expected to be greatest with elimination and IBE and least with stepwise backtracking. We intended to measure marker use for each participant via the average number of markers used per problem. This variable showed extremely high positive skew, since most participants did not use markers on any of the problems, but a small percentage used them heavily. Therefore, we used a binary variable (whether a participant used one or more markers versus no markers at all across all problems). The marker use data were consistent with these expectations. In studies 1, 2, and 3, respectively, 57, 72, and 68% of the participants that were classified as consistently using elimination used markers. In contrast, 21, 22, and 17% of consistent general backtracking users used markers, and 0, 7, and 11% of stepwise backtracking users used markers. The frequency of marker use was significantly higher in the elimination group than either of the backtracking groups (see “Appendix 2” for statistics). Also, 100, 55, and 80% of consistent IBE users used markers. The marker use data provides converging evidence for the strategy classifications.

### Predictors of strategy use (Q2)

We hypothesized that fluid intelligence and thinking dispositions would each account for unique variance in using elimination and IBE and correlate positively with these strategies. For Study 1, we tested these hypotheses with multiple regression, which was an adequate procedure since we used only one test to measure each predictor variable. For studies 2 and 3, we used structural equation modeling.

#### Study 1

We conducted two multiple regressions with elimination use and IBE use as separate outcome variables and SAT and open-mindedness as predictors. The mean SAT score was 1234 (SD 130). The mean open-mindedness score was 4.15 (SD = 0.40). SAT and open-mindedness scores were correlated, *r* = 0.291, *p* < 0.05. The regression analysis for elimination use was significant, *F*(2, 65) = 4.64, *p* = 0.013, *R*^2^ = 12.5% (adj. *R*^2^ = 9.8). The SAT score was positively associated with elimination use, *t* = 2.84, *p* < *0.0*1, but open-mindedness was not, *t* = 0.25, *p* = 0.81. SAT and open-mindedness uniquely accounted for 10.5% and 0.1%, respectively, of the variance in elimination use. Common variance accounted for 2.0%.

The regression analysis for IBE use was significant, *F*(2, 65) = 6.80, *p* = 0.002, *R*^2^ = 17.3% (adj. *R*^2^ = 14.7). SAT score was positively associated with IBE use, *t* = 2.91, *p* < 0.01, but open-mindedness was not, *t* = 1.31, *p* = 0.19. SAT and open-mindedness uniquely accounted for 9.6% and 2.2%, respectively, of the variance in IBE use. Common variance accounted for 5.4%. These analyses supported the hypothesis that use of elimination and IBE would correlate positively with fluid intelligence but did not support the hypothesis that these strategies would correlate with thinking dispositions.

#### Study 2

SAT, verbal analogies, and spatial reasoning scores were intended measures of intelligence. Typical Intellectual Engagement and Actively Open-minded-Thinking were intended measures of thinking dispositions. We had complete (*N* = 106) data for these variables except for nine missing SAT scores. These scores were imputed from the verbal analogies and transformed spatial reasoning scores using the Expectation–Maximization method (Gold and Bentler 2000). (When the analyses reported below were repeated without the nine participants with imputed SAT scores, the same pattern of significance and effect sizes occurred.) Because the spatial-reasoning scores showed a ceiling effect and high negative skew, an inverse transformation was applied. The outcome variables were elimination and IBE use. Tables 1 and 3 show descriptive statistics for the predictor and outcome variables, respectively. Table 4 shows the full correlation matrix.

**Table 3:** Descriptive statistics for predictor variables in Study 2

	*N*	*M*	SD	Skewness
SAT (400–1600 scale) with no imputing	97	1220	130	0.29
SAT with nine imputed scores	106	1217	128	0.31
Verbal analogies (*N* correct of 18)	106	11.0	3.00	0.02
Spatial reasoning (% correct)	106	96.5	7.8	− 3.51
Spatial reasoning transformed	106	0.804	0.270	− 1.01
Actively open-minded thinking (1–6 scale)	106	4.11	0.51	0.08
Typical intellectual engagement (1–6 scale)	106	3.87	0.63	− 0.46

*~ 0 to ~ 1.0 scale

**Table 4:** Correlations of predictors and dependent variables in Study 2

	Intelligence	Dispositions	Elimination use	IBE use
Intelligence (*F*1)	–			
Dispositions (*F*2)	0.483*	–		
Elimination use	0.583*	0.413*	–	
IBE use	0.676*	0.455*	0.697*	–
Stepwise	− 0.155	− 0.364*	− 0.531*	− 0.439*

*p < 0.05

Structural equation modeling was used to test whether the five predictor tests fit our hypothesized factor structure and then evaluate how well the hypothesized factors—fluid intelligence and thinking dispositions—predicted individual differences in use of elimination, IBE, and stepwise backtracking. We included in the same analysis outcome variables for all strategy use variables that were independently measured from the data—elimination use, IBE use, and percentage of stepwise backtracking tests—as opposed to being inferred using the classification scheme. Although we did not advance hypotheses about percentage of stepwise backtracking tests, we included it as an outcome variable because of its high correlation with elimination use. In an initial model including transformed spatial reasoning scores, spatial reasoning showed poor reliability as an indicator of intelligence (reliability = 0.08) and showed signs of multidimensionality through cross-loading. Due to this and the evidence of a ceiling effect, we removed spatial reasoning from the model. Keeping spatial reasoning in the model leads to the same pattern of effect sizes and significance.

**Fig. 4:**
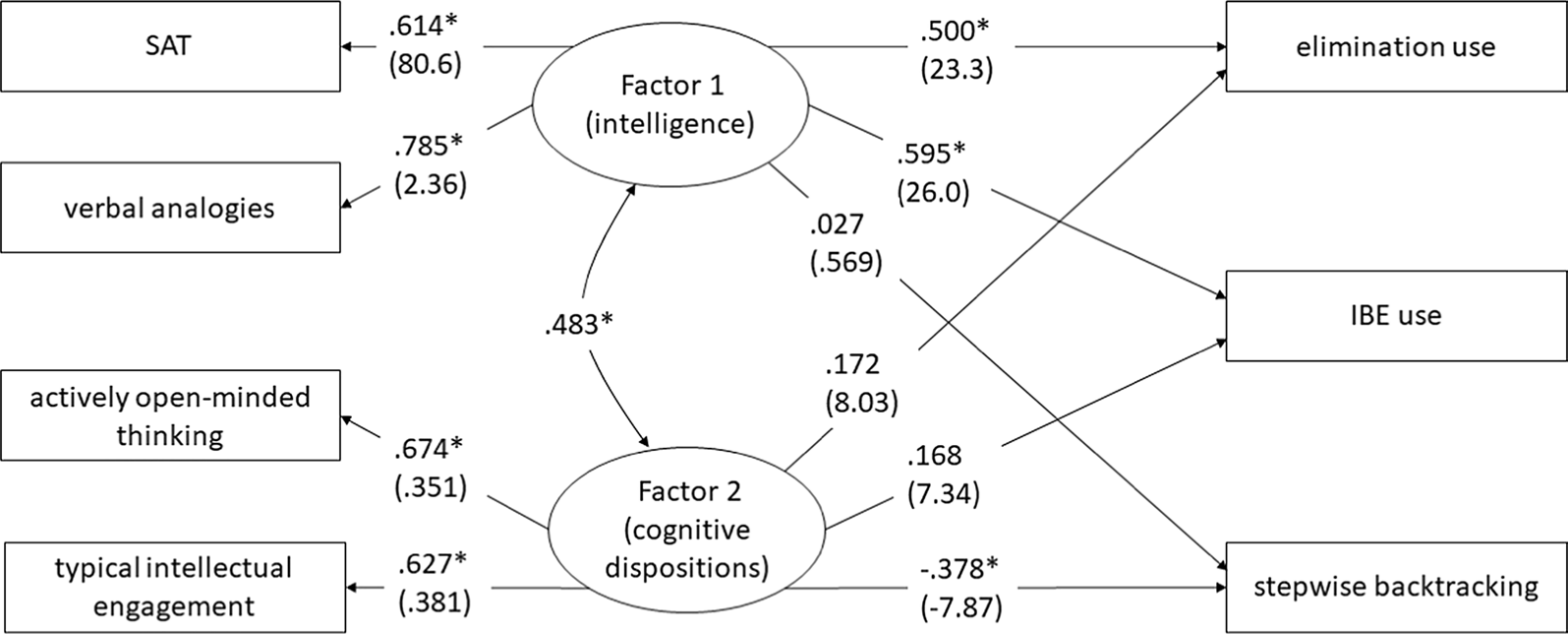
Structural Equation Model of the effect of intelligence and thinking dispositions on causal inferences in Study 2. Covariances among the three dependent variable error terms are omitted for clarity. Standardized solution (unstandardized coefficients in parentheses), *N* = 98. *p < 0.05

Fit for the final model was excellent: *X*^2^(5) = 11.05, *p* = 0.14, CFI = 0.979. RMSEA = 0.077. Factor loadings and standardized and unstandardized regression coefficients for all Study 2 variables except spatial reasoning are shown in Fig. 4. All predictors loaded 0.61 or higher on the hypothesized factors. Table 5 shows how the two factors accounted for unique and common variance in strategy use. Intelligence (F1) significantly predicted elimination and IBE use and uniquely accounted for large amounts of variance (> 25%) in each variable. Dispositions (F2) did not significantly predict elimination or IBE use and uniquely accounted for little variance (< 3%) in either. However, common effects of intelligence and dispositions accounted for 8–10% of variance in elimination and IBE.

**Table 5:** Predicting strategy use with intelligence (F1) and thinking dispositions (F2) in Study 2

	Beta			Variance components (*R*^2^ as proportion)			
	Intelligence	Dispositions	Error	Total	Intelligence unique	Dispositions unique	Common
Elimination use	0.500*	0.172	0.799	0.362	0.250	0.030	0.082
IBE use	0.595*	0.168	0.722	0.479	0.354	0.028	0.096
Stepwise backtracking	0.027	− 0.378*	0.931	0.133	0.001	0.143	− 0.010†

*p < 0.05

^†^Negative common variance may indicate slight suppression

Intelligence was not significantly related to stepwise backtracking use and accounted for very little variance in it. Interestingly, thinking dispositions showed a strong and significant *negative* association with stepwise backtracking use, uniquely accounting for 14% of the variance in the strategy. This fits with the finding by Sa et al. (2005) that participants with better thinking dispositions avoided poor argumentation techniques to a greater extent than those with lower dispositions.

#### Study 3

Because cognitive dispositions were not measured in Study 3, this study tested whether the findings of Study 2 regarding the intelligence predictor variable were replicated. SAT, verbal analogies, and paper folding were intended measures of fluid intelligence. Paper folding was scored in terms of number correct. Table 6 shows descriptive statistics for the predictor variables. We had complete (*N* = 105) data for these variables except for two missing SAT scores, which were imputed from the verbal analogies and paper folding scores as in Study 2. (The findings were the same when the two participants with imputed SAT scores were dropped.) The outcome variables were elimination, IBE, and stepwise backtracking use.

**Table 6:** Descriptive statistics for individual differences predictor variables in Study 3

	*N*	*M*	SD	Skewness
SAT (400–1600 scale)	103	1227	162	− 0.15
SAT with two imputed scores	105	1224	162	− 0.12
Verbal analogies (*N* correct of 18)	105	10.8	3.2	− 0.12
Paper folding (*N* correct of 20)	105	13.4	3.9	− 0.66

Structural equation modeling was used to determine the loadings of our three predictor tests on the (latent, reflective) construct of intelligence, as well as how intelligence predicted individual differences in strategy use. Fit for the model was very good: *X*^2^(6) = 10.88, *p* = 0.09, CFI = 0.943, RMSEA = 0.088 (see “Appendix 3”). Standardized loadings for intelligence on SAT, verbal analogies, and paper folding were 0.64, 0.58, and 0.76, respectively. Intelligence accounted for 34.4% of variance in elimination use and 43.1% of variance in IBE use. Both of these loadings were significant. In contrast, intelligence showed a nonsignificant and negative relationship with the use of stepwise backtracking, accounting for little variance (< 4%). These findings provided strong support for our individual-differences predictions regarding intelligence.

### Analytic versus heuristic processing (Q3)

The default-interventionist version of dual-processing theories predicts that analytic processing will be accurate and slow, whereas heuristic processing will be less accurate and fast. In the fault diagnosis problems, performance accuracy is indicated by minimizing the number of tests. We tested whether elimination and IBE showed characteristics of analytic processing—fewer but slower diagnostic tests—while backtracking showed characteristics of heuristic processing—more but faster tests. As all of the belief updating strategies discussed here are iterative, we used the time to complete one iteration, i.e., time per diagnostic test, as the measure of time use. The time for making each pipe test was measured from when the results of the previous test was displayed to when the participant clicked on a pipe or tank. Thus, the delays between initiating a test and seeing its result were excluded.

Table 7 shows data on number of tests per problem and time per test from the three studies for participants who were classified as consistently using different strategies. Participants made fewer tests during elimination (1.6–1.8) than during general (5.5–6.1) and stepwise (4.9–5.9) backtracking. Participants took at least five times longer, on average, to make diagnostic tests during elimination (27–41 s), as during general (5–7 s) and stepwise backtracking (3–5 s). These effects were large and significant (*d’*s > 2, see “Appendix 2”). Because elimination and the two backtracking strategies were measured using one set of problems and IBE using different problems, we did not statistically compare performance for the IBE group to the backtracking groups. However, like elimination, IBE users made few tests per problem (1.3–1.9) but slow tests (28–44 s). These data suggest that elimination and IBE have characteristics of analytic processing, whereas both backtracking strategies have characteristics of heuristic processing.

**Table 7:** Mean performance on the fault diagnosis task per problem by strategy group. Standard deviations in parentheses

Strategy group	Study 1			Study 2			Study 3		
	*N* tests	Time/test (s)	s/prob	*N* tests	Time/test (s)	s/prob	N tests	Time/test (s)	s/prob
Elimination	1.6 (0.3)	27.2 (12.0)	69 (30)	1.8 (0.4)	35.9 (16.8)	90 (23)	1.6 (0.4)	40.8 (23.9)	81 (34)
Transition	2.6 (0.5)	16.7 (5.5)	63 (14)	2.6 (0.8)	21.8 (19.8)	84 (27)	2.8 (0.9)	20.4 (14.6)	78 (39)
General backtracking	5.5* (2.0)	4.7* (2.3)	70 (45)	6.1* (1.6)	6.7* (4.3)	86 (36)	6.0* (1.6)	6.6* (4.1)	80 (35)
Stepwise backtracking	4.9* (0.5)	3.4* (2.5)	42* (17)	5.9* (0.7)	4.3* (2.0)	63* (17)	5.8* (0.8)	3.4* (1.8)	48* (23)
IBE	1.9 (0.1)	28.0 (6.2)	64 (12)	1.3 (0.3)	38.5 (16.9)	76 (16)	1.3 (0.2)	43.8 (16.4)	66 (21)

*p < 0.05 in comparison with elimination group

In addition, since analytic processes are defined as having higher working memory load than heuristic processes (Evans and Stanovich 2013) and marker use is an indication of working memory load, the finding reported above that elimination and IBE involved more marker use than backtracking provides further evidence for this conclusion. Table 7 shows that, over the course of a problem, the fewer tests made during elimination compensate for the longer time per test, so that time per problem is about the same with elimination and general backtracking. However, time per problem with stepwise backtracking is still faster than elimination.

One of our unexpected individual-differences findings was that, although the average time per test was much longer with elimination than stepwise backtracking, 14% of elimination users (across all three studies) made tests within the narrow range of fast times for stepwise-backtracking users. This finding is consistent with recent evidence that people sometimes give normative responses as fast as non-normative heuristic responses, as if they have automatized normative “intuitions” (Bago and De Neys 2017; Newman et al. 2017).

## General discussion

### Main findings

Our main research questions focused on whether normative fault diagnosis strategies were used infrequently and were correlated with fluid intelligence and thinking dispositions. We focused on strategies for updating beliefs about the fault set after observing new data.

#### Normative versus non-normative strategies

We showed that, by chaining diagnostic inferences from both normal (clean water) and abnormal (rusty) system observations, the practiced elimination strategy is normative—in the sense of maximizing information gain from each observation—and also minimizes the number of diagnostic tests^2^ required. IBE normatively maximizes information gain and minimizes number of tests when systems have multiple abnormal outputs that must be explained. In contrast to elimination, backtracking is inefficient because it ignores the valuable information from normal observations.

#### Individual-differences findings

As predicted, in three studies, elimination and IBE were used infrequently, while the less efficient stepwise and general backtracking were the modal strategies. Another study that found infrequent use of normative strategies during causal reasoning was Bramley et al. (2017). Using the task of learning causal structure by making interventions, they found that 7–20% of participants in two studies followed the prescriptions of a rational Bayes-optimal-observer model and were very accurate at determining structure.

As predicted, elimination and IBE use were strongly and positively correlated with fluid intelligence. In Study 2, where variance in intelligence could be partialled out from variance due to thinking dispositions, intelligence uniquely accounted for 25 and 35% of the variance in elimination and IBE use, respectively. Our prediction that thinking dispositions would account for unique variance in elimination and IBE use was not supported. However, in Study 2, variance shared by thinking dispositions and intelligence accounted for 8–10% of variance in elimination and IBE use. Furthermore, positive thinking dispositions were significantly associated with less use of the inefficient stepwise backtracking strategy, with dispositions accounting uniquely for 14% of variance in stepwise backtracking use. Thus, fluid intelligence and thinking dispositions were moderately to strongly associated with using normative fault diagnosis strategies and avoiding inefficient strategies.

These findings support studies using other reasoning tasks by Stanovich and West (1997, 1998); Toplak et al. (2014); and Klaczynski and Lavalee (2005) but extend the findings to a complex fault diagnosis task. These findings also fit with studies using a simpler diagnostic reasoning task by McNair and Feeney (2015) and Sirota et al. (2014). The association we found between positive thinking dispositions and less use of backtracking fits with Sa et al. (2005), who found that open-mindedness was associated with avoiding unsophisticated argumentation techniques. This result also supports Stanovich’s (2011, 2018) claim that thinking disposition tests assess ability at monitoring and overriding ineffective thinking. In general, our individual differences findings support a growing body of research showing that effective reasoning depends on non-intellective traits like self-discipline (Duckworth and Seligman 2005; Shoda et al. 1990) as well as on cognitive abilities.

#### Causal learning data

As noted earlier, in addition to the fault diagnosis task, participants in studies 2 and 3 also completed a causal learning task, which allowed us to investigate the generality of causal reasoning strategies. Since effective fault diagnosis depends on accurate causal models, the quality of individuals’ causal learning should correlate positively with the quality of their diagnostic reasoning. Instead of learning from interventions, our participants observed data on co-occurrences of a candidate causal variable and its effects and then made structure judgments (by estimating their confidence that a causal link existed) and causal strength judgments (Lu et al. 2008). As with the fault diagnosis task, we investigated the frequency and the cognitive correlates of using normative strategies. We plan to report these findings in a follow-up paper. Preliminary findings from the causal learning task (Shreeves et al. 2018) were similar to the fault diagnosis findings. Relatively few participants made normative strength judgments, as defined by the causal power model (Lu et al. 2008), or normative confidence judgments, as defined by Griffiths and Tenenbaum’s (2005) causal support model. Greater fluid intelligence was significantly associated with making normative strength and confidence judgments. To our knowledge, no studies have compared the performance of the same participants on both fault diagnosis and causal learning tasks.

#### Analytic versus heuristic processing

Regarding our third research question, elimination and IBE users made fewer but slower tests compared to backtracking users. This finding supports the predictions of default–interventionist dual processing theories and provides strong evidence that elimination and IBE involve analytic processing while backtracking is heuristic. Similarly, Bramley et al. (2017) claimed that the infrequently used normative procedure for selecting interventions (which was discussed above) heavily loads working memory. Thus, this procedure probably involves analytic processing. In contrast, their other participants used heuristics, which reduced memory load during causal learning.

In addition, one of our individual-differences findings supports recent theories that critique the default–interventionist view of dual processing (e.g., Newman et al. 2017). A small percentage of elimination users made normative responses but did so as quickly as the much faster backtracking users. These participants seemed to be able to quickly transfer prior knowledge of the elimination strategy to the fault diagnosis task.

### Implications for fault diagnosis

The literature review and our analysis of fault diagnosis strategies suggested that when they are faced with complex problems, people use qualitative, hypothetical reasoning when updating fault hypotheses based on evidence. We pointed out that making predictive inferences from hypotheses was important in discovering the elimination strategy and using IBE. Waldman and Hagmayer (2005) suggest that predictive inferences like these are a hallmark of causal reasoning and provide empirical evidence that people make predictive inferences when updating their causal beliefs based on evidence.

A limitation of the current studies is that we explicitly instructed our participants about the single-fault constraint. One could argue that, because of this, we should not have labeled participants who met our operational definition of IBE (based on Eq. 2) as IBE users. In the introduction, we argued that IBE goes beyond the simplicity (single-fault) assumption. IBE involves making inferences to identify the explanation that best balances criteria including simplicity, coverage (maximizing the effects explained), and coherence with background information. Our results demonstrate that the participants we classified as IBE users could make the normative inferences needed to identify the explanation that maximized both simplicity and coverage, while other participants did this less well. Thus, we feel that the IBE label is appropriate, although we admit that our evidence for IBE use would be stronger if we had not explicitly communicated the single-fault constraint and that the infrequent use of IBE in the current studies might be even lower if this constraint had not been mentioned.

#### Logical versus probabilistic reasoning

Although we have characterized fault diagnosis as involving inductive, probabilistic reasoning, an argument can be made that fault diagnosis of deterministic systems involves deductive logic. In probabilistic systems, network components function as probabilistic, noisy-OR gates (Lu et al. 2008); but in deterministic systems like the tank networks, components function as logical-OR gates (always outputting rusty water if they are rusty *or* get rusty input water). This argument follows Oaksford and Chater (2012), who pointed out that deductive logic is a special case of probabilistic reasoning. We agree with their proposal that human deductive and inductive reasoning can be accounted for by a unitary probabilistic reasoning system that makes different assumptions (e.g., deterministic causal strengths in a causal model) in different task contexts. We make no claim regarding whether the participants in our task were using inductive or deductive reasoning.

However, researchers have found evidence for deterministic, qualitative reasoning in tasks like medical diagnoses, where deterministic assumptions are much less viable than in our task (Eddy and Clanton 1982; Rossi and Madden 1979; Yeung and Griffiths 2015). Our literature review suggests that physicians use practiced elimination and IBE. Using deterministic strategies in probabilistic domains can lead to errors. We see two ways that diagnosticians can benefit from deterministic reasoning without making too many errors. First, they can follow the practice that Payne and Bettman (2004) observed in research on decision-making, where participants sometimes used the qualitative elimination-by-aspects strategy to narrow a set of decision alternatives and then switched to the quantitative weighted-additive strategy on the simpler problem. Second, they can use deterministic reasoning in a defeasible manner, switching to quantitative reasoning if deterministic reasoning leads to anomalies.

### Applications

Our evidence suggesting that a small percentage of participants showed fast but normative diagnosis performance highlights the importance of learning effective diagnosis strategies. When fault diagnosis is viewed as finding the causes of specific problems, strategies like elimination and IBE are applicable in diagnosing disease and equipment malfunctions but also in scientific, legal, and political argumentation. Given this wide applicability, training fault diagnosis strategies may improve reasoning in a variety of domains. Prior research on improving fault diagnosis by explicit strategy training has taught either domain-specific strategies that seem unlikely to transfer to new situations (Kostopoulo and Duncan 2001) or backtracking strategies that do not minimize tests (Carlson et al. 1992). On average, elimination and IBE users minimized diagnostic tests at the cost of extra time, whereas stepwise backtracking users reduced time per test and per problem at the cost of excessive tests. This suggests that people should be taught a repertoire of fault diagnosis strategies. The extra time it takes to use elimination and IBE may be worthwhile if testing costs are high. Stepwise backtracking may be the best choice under time pressure, when testing costs are low or for people with lower fluid intelligence. This idea is consistent with research showing that people adaptively choose decision-making strategies to meet changing task constraints (Gigerenzer and Todd 1999; Payne and Bettman 2004).

Because strategies like elimination and IBE are useful for understanding policy issues (e.g., causes of global warming or violent crime), our findings are relevant to teaching critical thinking to the public. We found that fluid intelligence and thinking dispositions were positively associated with using elimination and IBE. Since thinking dispositions may be more trainable than intelligence (Klaczynski and Lavalee 2005), training in thinking dispositions may be the more effective way to indirectly increase use of elimination and IBE. Books on improving thinking skills often emphasize explicit teaching of thinking dispositions (Stanovich 2013; Kuhn 2005). A more direct route to improving critical thinking is to teach reasoning strategies explicitly, as is done in in books on improving thinking (Kuhn 2005; Nisbett 2015). An interesting task for future research is to facilitate use of effective fault-diagnosis strategies either directly or indirectly.

## Figures and Tables

**Fig. 5:**
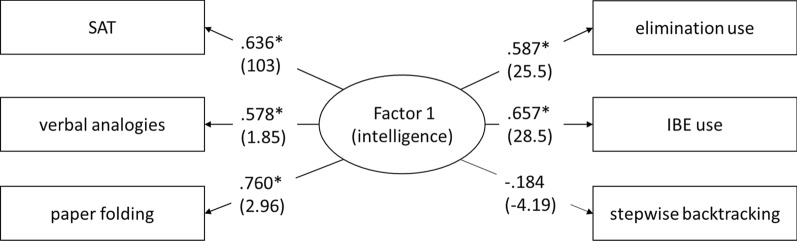
Structural Equation Model of the effect of intelligence on causal inferences. Fit was very good, *X*^2^(6) = 10.88, *p* = .09, CFI = 0.943, RMSEA = 0.088. Covariances among the dependent variable error terms are omitted for clarity (See Table 11). Standardized solution (unstandardized coefficients in parentheses), *N* = 105. *p < 0.05

## Data Availability

The datasets used and/or analyzed during the current study are available from the corresponding author on reasonable request.

## Appendix 1: Tank networks

The 18 tank networks used in Study 1, in order of presentation. The rusty tank is indicated by a square. Rusty water output from the network is indicated by an R. All other network inputs and outputs were clean (C). The first nine networks have one rusty network output, the second nine have two. Networks 3, 6, 10, 12, 13, 21, 22, 25, 27, and 29 were used in studies 2 and 3.

## Appendix 2: Statistical comparison of strategy use groups on performance variables

This appendix presents the statistical analyses of the performance of participants who used different strategies that were discussed in the Results section under the heading “Analytic versus heuristic processing.” Participants who were classified as transitioning between backtracking and elimination strategies were excluded from these comparisons as our expectations about the differences between analytic versus heuristic processing applied only to backtracking and elimination (Tables 8, 9, 10).

**Table 8:** Marker use by strategy group in study 1

	Stepwise backtracking	General backtracking	High elimination
Did not use markers	17	27	6
Used any markers	0	7	8

**Table 9:** Marker use by strategy group in Study 2

	Stepwise backtracking	General backtracking	High elimination
Did not use markers	13	36	5
Used any markers	1	10	13

**Table 10:** Marker use by strategy group in Study 3

	Stepwise backtracking	General backtracking	High elimination
Did not use markers	16	35	6
Used any markers	2	7	13

### Study 1

#### Number of diagnostic (pipe) tests

The number of pipe tests was approximately normally distributed but displayed heteroscedasticity (Levene’s test (2, 62) = 12.35, *p* < 0.001). An ordinary least squares one-way ANOVA was conducted to determine the effect of group membership on number of pipe tests, followed by a weighted least squares analysis. Groups significantly differed in pipe test usage, *F* (2, 62) = 37.13, *p* < 0.001, *η*^2^ = 0.545. Bonferroni-corrected post-hoc comparisons showed that those in the high elimination group made significantly fewer pipe tests (*M* = 1.59, *SE* = 0.074) than those using general backtracking, mean difference = 3.96, *p* < 0.001, CI_.95_ [− 5.08, − 2.84], *d* = 2.4, and those using stepwise backtracking, mean difference = 3.35, *p* < 0.001, CI_.95_ [− 4.32, − 2.08], *d* = 8.2. The number of pipe tests did not differ significantly between stepwise and general backtracking users, *p* = 0.349. (The same pattern of significance occurred using weighted least squares.)

#### Time per test

Time per test and completion time were highly positively skewed in each condition, and simple transformations were not able to account for both skew and kurtosis. Gamma-distribution generalized linear models were used for group comparisons instead of general linear models for these variables. One participant was excluded as a multivariate outlier in time per test. A likelihood ratio test for the gamma distribution with an inverse link provided evidence for a significant difference between the groups, *F* (2, 63) = 114.10, *p* < 0.001, *R*^2^_*L*_ = 0.592. The high elimination use group (predicted value = 27.23 s) had significantly longer times per test than those in the stepwise backtracking group (predicted value = 2.83 s), *t* (63) = 203.84, *p* < 0.001, *d* = 3.4, and those in the general backtracking group (predicted value = 3.65 s), *t* (63) = 164.71, *p* < 0.001, *d* = 2.9.

#### Marker use

Most participants used no markers, and data for participants using any number makers more than zero were insufficient to approximate a normal distribution under any transformation. Additionally, extremely unequal variances between groups, including a variance of 0 in the stepwise backtracking group (as nobody in this group used markers), prevented mean comparisons. Participants were coded as either using any markers or no markers at all, and a Fisher’s exact test was conducted to determine if marker use was dependent on strategy group. See Table 8 for marker use. Marker usage was significantly different between groups, *p* < 0.001. When a Bonferroni corrected alpha level of 0.025 was used for post-hoc tests, those in the high elimination group were significantly more likely to use markers than those who used stepwise backtracking, *p* < 0.001, and those who used general backtracking, *p* = 0.019.

#### Time per problem

One additional participant was excluded from the time per problem data as a multivariate outlier, resulting in 64 participants. A likelihood ratio test for the gamma distribution with an inverse link provided evidence for a significant difference between the groups, *F* (2, 63) = 13.18, *p* = 0.001, *R*^2^_*L*_ = 0.17. The high elimination use group (predicted value = 68.74 s) had significantly longer completion times than those in the stepwise backtracking group (predicted value = 42.50 s), *t* (63) = 11.701, *p* = 0.001, *d* = 1.1, but not those in the general backtracking group (predicted value = 63.96 s), *t* (63) = 0.336, *p* = 0.562, *d* = 0.03.

### Study 2

#### Number of diagnostic (pipe) tests

The number of pipe tests was approximately normally distributed but displayed heteroscedasticity (Levene’s test (2, 75) = 6.23, *p* = 0.003). An ordinary least squares one-way ANOVA was conducted to determine the effect of group membership on number of pipe tests, followed by a weighted least squares analysis. Groups significantly differed in pipe test usage, *F* (2, 75) = 82.14, *p* < 0.001, *η*^2^ = 0.687. Bonferroni-corrected post-hoc comparisons showed that those in the high elimination group made significantly fewer pipe tests (*M* = 1.76, *SE* = 0.30) than those using general backtracking, mean difference = − 4.35, *p* < 0.001, CI_.95_ [− 5.19, − 3.53], and those using stepwise backtracking, mean difference = − 4.11, *p* < 0.001, CI_.95_ [− 5.18, − 3.05]. The number of pipe tests did not differ significantly between stepwise and general backtracking users, *p* = 0.796. (The same pattern of significance occurred using weighted least squares.)

#### Time per test

Time per pipe test in Study 2 was highly positively skewed, so a gamma-distribution generalized linear model was used. A likelihood ratio test for the gamma distribution with an inverse link provided evidence for a significant difference between the groups, *F* (2, 73) = 103.70, *p* < 0.001, *R*^2^_*L*_ = 0.44. Furthermore, the high elimination use group (predicted value = 35.90 s) had significantly longer times per test than those in the stepwise backtracking group (predicted value = 4.26 s), *t* (73) = 125.93, *p* < 0.001, and the general backtracking group (predicted value = 6.69 s), *t* (73) = 128.48, *p* < 0.001.

#### Marker use

Participants were coded as either using any markers or no markers at all (see Table 9 for marker use). Unlike that observed in Study 1, sufficient variance was present to conduct a binary logistic generalized linear model to compare marker usage among groups. Marker usage was significantly different between groups, *F* (76, 2) = 19.65, *p* < 0.001, *R*^2^_*L*_ = 0.204. Post-hoc comparisons using least significant difference found that those in the high elimination group were significantly more likely to use markers (72%) than those who used stepwise backtracking (7%), *t* (76) = 9.154, *p* = 0.002, and those who used general backtracking (22%), *t* (76) = 12.359, *p* = 0.019.

#### Time per problem

Three additional participants were eliminated from the time per problem analysis as multivariate outliers. An ordinary least squares one-way ANOVA was conducted to determine the effect of group membership on completion time. Groups significantly differed in time per problem, *F* (2, 72) = 5.01, *p* = 0.009, *η*^2^ = 0.122. Bonferroni-corrected post-hoc comparisons showed that those in the high elimination group took significantly longer to complete tasks than those using stepwise backtracking, mean difference = 26.82, *p* = 0.007, CI_.95_ [6.03, 47.62], but not those using general backtracking, mean difference = 10.91, *p* = 0.321, CI_.95_ [− 5.48, 27.29]. Time per problem did not differ significantly between stepwise and general backtracking users, *p* = 0.099.

### Study 3

#### Number of diagnostic (pipe) tests

The number of pipe tests was approximately normally distributed but displayed heteroscedasticity (Levene’s test (2, 76) = 6.01, *p* = 0.004). An ordinary least squares one-way ANOVA was conducted to determine the effect of group membership on the number of pipe tests, followed by a weighted least squares analysis. Groups significantly differed in pipe test usage, *F* (2, 76) = 82.86, *p* < 0.001, *η*^2^ = 0.686. Bonferroni-corrected post-hoc comparisons showed that those in the high elimination group made significantly fewer pipe tests than those using general backtracking, mean difference = − 4.35, *p* < 0.001, CI_.95_ [− 5.19, − 3.51], and those using stepwise backtracking, mean difference = − 4.13, *p* < 0.001, CI_.95_ [–5.13, − 3.14]. The number of pipe tests did not differ significantly between stepwise and general backtracking users, *p* = 0.820. (The same pattern of significance occurred using weighted least squares, *F* (2, 76) = 285.69, *p* < 0.001, *η*^2^ = 0.883.)

#### Time per test

The time per pipe test in Study 3 was highly positively skewed, so a gamma-distribution generalized linear model was used. A likelihood ratio test for the gamma distribution with an inverse link provided evidence for a significant difference between the groups, *F* (2, 77) = 113.00, *p* < 0.001, *R*^2^_*L*_ = 0.434. Furthermore, the high elimination use group (predicted value = 40.84 s) had significantly longer times per test than the stepwise backtracking group (predicted value = 3.38 s), *t* (77) = 169.41, *p* < 0.001, and the general backtracking group (predicted value = 6.68 s), *t* (77) = 128.72, *p* < 0.001.

#### Marker use

Participants were coded as either using any markers or no markers at all (see Table 10 for marker use). Unlike that observed in Study 1, sufficient variance existed to conduct a binary logistic generalized linear model to compare marker usage among groups. Marker usage was significantly different between groups, *F*(77.2) = 19.36, *p* < 0.001, *R*^2^_*L*_ = 0.208. When a Bonferroni corrected alpha level of 0.025 was used for post-hoc tests, those in the high elimination group were significantly more likely to use markers (68%) than those who used stepwise backtracking (11%), *t*(77) = 10.10, *p* = 0.001, and those who used general backtracking (17%), *t*(77) = 13.68, *p* < 0.001.

#### Time per problem

One additional participant was excluded as a multivariate outlier. An ordinary least squares one-way ANOVA was conducted to determine the effect of group membership on completion time. Groups significantly differed in time per problem, *F* (2, 75) = 6.76, *p* = 0.002, *η*^2^ = 0.153. Bonferroni-corrected post-hoc comparisons showed that those in the high elimination group took significantly longer to complete tasks than those using stepwise backtracking, mean difference = 33.11, *p* = 0.001, CI_.95_ [10.89, 55.32], but not those using general backtracking, mean difference = 2.37, *p* = 0.99, CI_.95_ [− 27.21, 31.96]. The time per problem did not differ significantly between stepwise and general backtracking users, *p* = 0.087.

## Appendix 3: Structural equation modeling diagram and correlations for study 3

See Fig. 5 and Table 11.

**Table 11:** Correlations between observed variables in Study 3, with standard deviation on the diagonal. Estimated correlations between dependent variables included in the model are in parentheses

	SAT	Verbal analogies	Paper-fold	Elimination	IBE	Stepwise
SAT (imputed)	162					
Verbal analogies	0.481	3.20				
Paper folding	0.457	0.404	3.90			
Elimination use	0.287	0.334	0.500	43.4		
IBE use	0.366	0.326	0.556	0.749 (0.595)	43.4	
Stepwise	0.015	− 0.106	− 0.218	− 0.518 (− 0.515)	− 0.444 (− 0.436)	22.8

*N* = 105

## Abbreviation

IBE: Inference to the best explanation

## Footnotes

1 Here, general-purpose only means applicable across knowledge domains. We make no claims about whether fault diagnosis knowledge is represented in memory in a general or domain-specific manner.

2 Money spent is not the best measure of whether elimination and IBE users approached normative performance levels because minimizing the money spent requires both minimizing diagnostic tests and making a single diagnosis. The latter variable was not expected to be affected by elimination and IBE. Money spent showed the same pattern of effects as the number of diagnostic tests.
